# Examining the cross-sectional relationship of platelet/high-density lipoprotein cholesterol ratio with depressive symptoms in adults in the United States

**DOI:** 10.1186/s12888-024-05878-x

**Published:** 2024-06-07

**Authors:** Junjie Ni, Pu Wu, Xiaofeng Lu, Chaoyang Xu

**Affiliations:** 1 Department of Breast and Thyroid Surgery, Affiliated Jinhua Hospital, Zhejiang University School of Medicine, No. 365 Renmin East Road, Wucheng District, Jinhua, Zhejiang Province 321000 China; 2 Central Laboratory, Affiliated Jinhua Hospital, Zhejiang University School of Medicine, Jinhua, Zhejiang Province China

**Keywords:** Platelet/high-density lipoprotein cholesterol ratio, Depression, NHANES, United States adults, Cross-sectional study

## Abstract

**Aims:**

Herein, we examined the correlation between platelet/high-density lipoprotein cholesterol ratio (PHR) and symptoms of depression among United States adults.

**Methods:**

Data acquired from the 2007–2018 National Health and Nutrition Examination Survey, involving individuals ≥ 20 years of age, with available PHR and depression diagnosis information. We employed weighted uni- and multivariable logistic regression analyses to assess the distinct correlation between PHR and depressive symptoms. Additionally, we conducted subgroup, interaction, and restricted cubic spline analyses.

**Results:**

In all, 28,098 subjects were recruited for analysis, with 8.04% depression status and 19.31 ± 0.11 mean PHR value. Depressive symptoms increased with higher quartiles of PHR. Following fully confounder adjustments in model 2, participants with the largest PHR quartiles exhibited a 53% (OR: 1.53, 95%CI: 1.00–2.33, *P* = 0.05) raised depressive symptoms, relative to participants with least PHR quartiles. Based on the two-piece-wise regression, the breakpoint was PHR = 23.76, and a positive association was more evident when PHR < 23.76 (OR = 1.06, 95%CI: 1.02–1.10, *P* = 0.01). When PHR ≥ 23.76, the correlation disappeared (*P* = 0.85). Using subgroup and interaction analyses, we revealed a positive relationship between PHR and depressive symptoms almost consistent among various population settings.

**Conclusions:**

A convenient biomarker, the PHR was independently associated with an increased risk of depressive symptoms and may be a promising new bioindicator for the prediction of depression diagnosis.

**Supplementary Information:**

The online version contains supplementary material available at 10.1186/s12888-024-05878-x.

## Introduction

Depression is a globally widespread phenomenon which accompanies disinterest in daily activities, insomnia, loss of life pleasures, and emergence of suicidal interests. This condition influences both mental and physical health [1]. Statistics from the global burden of disease revealed a sharp rise (49.86%) in global depression incidences from 172 million in 1990 to 258 million in 2017 [2]. Moreover, based on World Health Organization (WHO) reports, depression currently ranks third among the leading causes of the global disease burden, and it is projected to become the foremost cause of burden of disease by 2030 due to its high incidence and its significant contribution to increased risk of disability and mortality [1]. Regrettably, even in the advent of new anti-depression therapies, the depressive recurrence rate remains elevated, and close to 80% patients experience a second episode [3]. Hence, it is critical to establish novel bioindicators of depression risk to enhance depression prevention and intervention.

Till date, there is no consensus on depression pathophysiology. However, emerging evidences suggest a strong role of inflammation in depression etiology [4, 5]. For example, one study showed that pro-inflammatory cytokine administration in a clinical setting augments depressive symptoms [6]. Research has also indicated that immune mediators might influence neurotransmission, neural activity, and neuroendocrine pathways, potentially contributing to major depression [7]. Moreover, based on certain clinical investigations, individuals suffering from autoimmune diseases and diabetes are more prone to developing depression [8–10]. Patients with depression often exhibit marked alterations in the peripheral and/or central inflammation [11, 12]. Moreover, several studies employed anti-inflammatory intervention to reduce depressive symptoms [13]. Thus, the role of inflammation in depression has garnered considerable attention. Some investigations have been conducted into the correlation between depression and inflammatory indicators like C-reactive protein (CRP), tumor necrosis factor-alpha (TNF-a), and interleukin-6 (IL-6) [14–16]. Considering these evidences, there is a clear association between inflammation and depression that can potentially be harnessed to treat or prevent depression.

The platelet/high-density lipoprotein cholesterol ratio (PHR) is a relatively new integrated assessment of inflammatory and hypercoagulability markers [17, 18]. Platelets release proinflammatory cytokines which promote the emergence and progression of inflammatory diseases. Upregulated and active platelets potentially give rise to, sustain, and regulate inflammatory responses among depressed patients [19]. Dietrich-Muszalska et al. demonstrated that active platelets increase inflammation-mediated blood platelet accumulation among patients suffering from mental disease [20]. High-density lipoprotein cholesterol (HDL-C) protects against atherogenesis and oxidative stress among cardiovascular disease patients [21], in addition to its role in anti-platelet, other anti-thrombotic [22, 23], and anti-inflammatory activities [24]. Nonetheless, there is much controversy in the potential HDL-C and depression link. One prospective investigation involving 246 subjects revealed that the HDL-C content is inversely proportional to depression severity [25]. Maes and colleagues discovered that lower serum HDL-C levels could serve as markers for major depression and suicidal behavior in depressed men, likely induced by the immune/inflammatory response in depression and associated with impaired reverse cholesterol transport from body tissues to the liver [26]. Conversely, a cross-sectional investigation reported a strong positive correlation between enhanced HDL-C levels and depression in both genders [27]. Moreover, a meta-analysis uncovered a correlation between high levels of HDL-C and increased depression levels, particularly among women [28]. Until now, there are no reports evaluating the association between PHR and depression. Therefore, as a newly defined systematic inflammatory marker, the PHR significance in depression requires additional exploration.

In this report, we hypothesized a possible relationship between PHR and depressive symptoms. To assess this relationship, we employed a large patient population with extensive relevant data, and adjusted for confounders, as appropriate. The conclusions of this study will enhance the mechanistic knowledge behind the inflammatory association of depression, as well as identification of robust bioindicators of this life-altering condition.

## Materials and methods

### Sample population and data acquisition

**Fig. 1:**
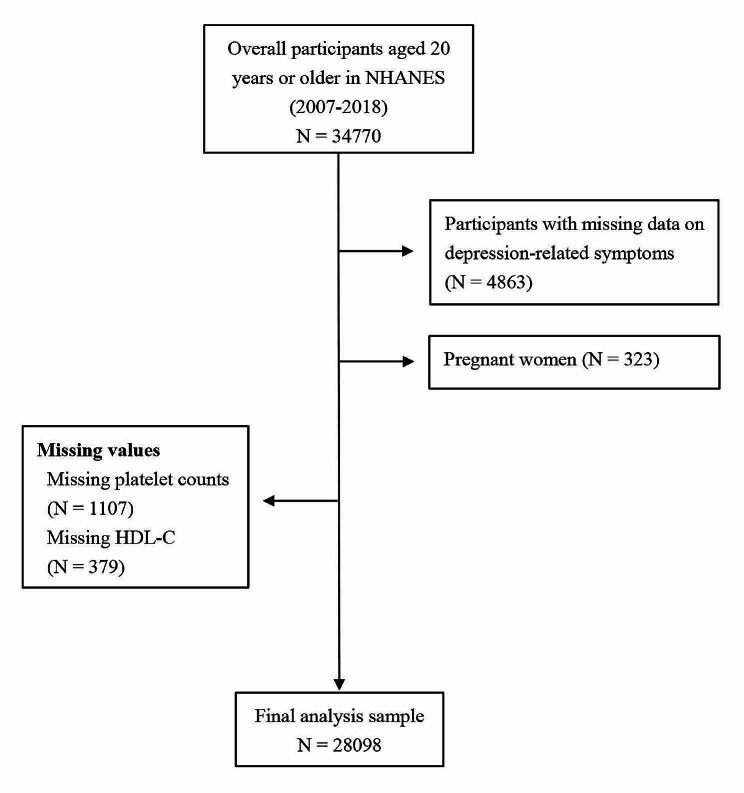
Representation of study design. *Abbreviations * NHANES, National Health and Nutrition Examination Survey; HDL-C, High-density lipoprotein cholesterol

To conduct this retrospective analysis, data was sourced from the 2007–2018 National Health and Nutrition Examination Survey (NHANES, CDC, https://www.cdc.gov/nchs/nhanes/index.htm). NHANES utilizes an extensive, multistep, probability-clustering technique to obtain information for evaluation regarding the health and nutritional distribution among US adults and children. Its sampling approach utilized subjected from varied racial and ethnic backgrounds, including non-Hispanic black, non-Hispanic white, Mexican American, and so on. The overall participants in NHANES were 34,770 individuals ≥ 20 years of age. For this investigation, we eliminated some subjects as follows: those with missing depression-related symptoms data (*n* = 4863), pregnant women (*n* = 323), unavailable platelet counts (*n* = 1107), and missing HDL-C contents (*n* = 379). Following screening based on our strict inclusion and exclusion guidelines, we were left with 28,098 study subjects (Fig. 1). NHANES procedure received authorization from the National Center for Health Statistics (NCHS) Ethics Committee, and acquired informed consent from subjects prior to the initiation of study [29]. All acquired data were from the aforementioned open access database and can be accessed from other sources as well [30].

## Results

### Descriptive analysis

The clinical and biochemical profiles of subjects with/without elevated depressive symptoms were presented in Table 1. In all, 28,098 subjects were recruited for analysis, with 47.91 ± 0.24 years average age, 19.31 ± 0.11 average PHR index, average overall depression prevalence of 8.03% and increased with the rising PHR quartiles (Quartile 1: 5.99%, Quartile 2: 7.72%, Quartile 3: 8.69%, Quartile: 9.81%, Supplementary Table 1). In individuals with elevated or non-elevated depressive symptoms, except for age, LDL-C and fasting TC (*P* > 0.05), marked alterations were observed in platelet counts, HDL-C contents, gender, race, marital status, education level, family-PIR, BMI, diet status, smoking status, alcohol usage, vigorous recreational activities, sleep disorder, DM, hypertension, CKD and ASCVD (all *P* < 0.05).

**Table 1:** Initial descriptions of participants between with and without elevated depressive symptoms from the 2007–2018 cycles

Characteristics (weighted)		Elevated depressive symptoms		*P*-value
	Total (*N* = 28,098)	No (*N* = 25,508)	Yes (*N* = 2590)	
Age (years)	47.91 ± 0.24	47.97 ± 0.26	47.17 ± 0.46	0.12
PHR continuous	19.31 ± 0.11	19.19 ± 0.12	20.71 ± 0.23	< 0.0001
Platelet (1000cells/uL)	243.43 ± 0.85	242.59 ± 0.87	253.02 ± 1.99	< 0.0001
HDL-C (mmol/L)	1.38 ± 0.01	1.38 ± 0.01	1.33 ± 0.01	< 0.0001
LDL-C (mmol/L)	2.94 ± 0.01	2.94 ± 0.01	2.98 ± 0.04	0.32
Fasting TC (mmol/L)	5.00 ± 0.01	4.99 ± 0.01	5.06 ± 0.04	0.05
PHR, n (%)				< 0.0001
Q1	7027(25.37)	6534(25.93)	493(18.94)	
Q2	7018(25.57)	6406(25.66)	612(24.59)	
Q3	7030(24.92)	6347(24.74)	683(26.98)	
Q4	7023(24.13)	6221(23.67)	802(29.49)	
Sex, n (%)				< 0.0001
Male	13,977(49.37)	13,028(50.52)	949(36.13)	
Female	14,121(50.63)	12,480(49.48)	1641(63.87)	
Race, n (%)				< 0.001
Mexican American	4287(8.53)	3892(8.55)	395(8.27)	
Non-Hispanic Black	5754(10.44)	5218(10.25)	536(12.69)	
Non-Hispanic White	11,898(67.73)	10,803(68.11)	1095(63.42)	
Other race	6159(13.30)	5595(13.09)	564(15.63)	
Marital status, n (%)				< 0.0001
Married	16,689(63.41)	15,536(64.81)	1153(47.39)	
Live separated	6303(18.49)	5406(17.39)	897(31.09)	
Never married	5093(18.07)	4556(17.78)	537(21.42)	
Missing	13(0.03)	10(0.02)	3(0.10)	
Education level, n (%)				< 0.0001
Less than high school	2787(4.94)	2403(4.63)	384(8.50)	
High school	10,332(33.37)	9182(32.52)	1150(43.15)	
More than high school	14,958(61.64)	13,904(62.81)	1054(48.28)	
Missing	21(0.04)	19(0.04)	2(0.07)	
Family PIR, n (%)				< 0.0001
< 1	5358(13.01)	4476(11.81)	882(26.80)	
1–3	10,931(33.50)	9865(32.82)	1066(41.40)	
> 3	9328(46.42)	8935(48.32)	393(24.59)	
Missing	2481(7.06)	2232(7.05)	249(7.21)	
BMI (kg/m^2, n (%))				0.01
<25	7778(28.77)	7180(29.05)	598(25.61)	
≥25	20,061(70.55)	18,106(70.30)	1955(73.45)	
Missing	259(0.67)	222(0.65)	37(0.94)	
Smoke status, n (%)				< 0.0001
Never	15,479(55.22)	14,423(56.68)	1056(38.51)	
Former	6877(25.17)	6286(25.39)	591(22.60)	
Now	5725(19.57)	4782(17.88)	943(38.90)	
Missing	17(0.04)	17(0.04)	0(0.00)	
Alcohol usage, n (%)				< 0.0001
Never	3869(10.47)	3550(10.59)	319(9.19)	
Former	4240(12.31)	3706(11.79)	534(18.32)	
Moderate	6722(27.52)	6104(27.54)	618(27.32)	
Heavy	5546(20.97)	4923(20.59)	623(25.36)	
Missing	7721(28.72)	7225(29.50)	496(19.81)	
Vigorous recreational activities, n (%)				< 0.0001
No	21,913(73.73)	19,597(72.47)	2316(88.24)	
Yes	6185(26.27)	5911(27.53)	274(11.76)	
Diet status, n (%)				0.01
Needs improvement	26,476(95.13)	24,033(95.14)	2443(95.08)	
Good diet	624(2.16)	590(2.24)	34(1.26)	
Missing	998(2.71)	885(2.62)	113(3.66)	
Sleep disorder, n (%)				< 0.0001
No	17,292(58.88)	15,928(59.76)	1364(48.85)	
Yes	1669(5.82)	1237(4.93)	432(16.04)	
Missing	9137(35.29)	8343(35.31)	794(35.12)	
DM, n (%)				< 0.0001
No	22,539(85.16)	20,655(85.70)	1884(78.90)	
Yes	5559(14.84)	4853(14.30)	706(21.10)	
Hypertension, n (%)				< 0.0001
No	16,240(62.87)	14,985(63.74)	1255(52.93)	
Yes	11,857(37.13)	10,522(36.26)	1335(47.07)	
Missing	1(0.00)	1(0.00)	0(0.00)	
CKD, n (%)				< 0.001
No	22,730(84.71)	20,740(85.00)	1990(81.38)	
Yes	5105(14.49)	4530(14.21)	575(17.66)	
Missing	263(0.80)	238(0.79)	25(0.97)	
ASCVD, n (%)				< 0.0001
No	25,244(91.86)	23,113(92.44)	2131(85.15)	
Yes	2849(8.13)	2391(7.55)	458(14.82)	
Missing	5(0.01)	4(0.01)	1(0.03)	

Continuous data are shown as as means and standard error (SE), while categorical data are presented as percentages. Abbreviations: HDL-C, high-density lipoprotein cholesterol; LDL-C, low-density lipoprotein cholesterol; TC, fasting total cholesterol; PIR, poverty income ratio; BMI, body mass index; PHR, platelet/high-density lipoprotein cholesterol ratio; CKD, chronic kidney disease; DM, diabetes mellitus; ASCVD, arteriosclerotic cardiovascular disease

**Fig. 2:**
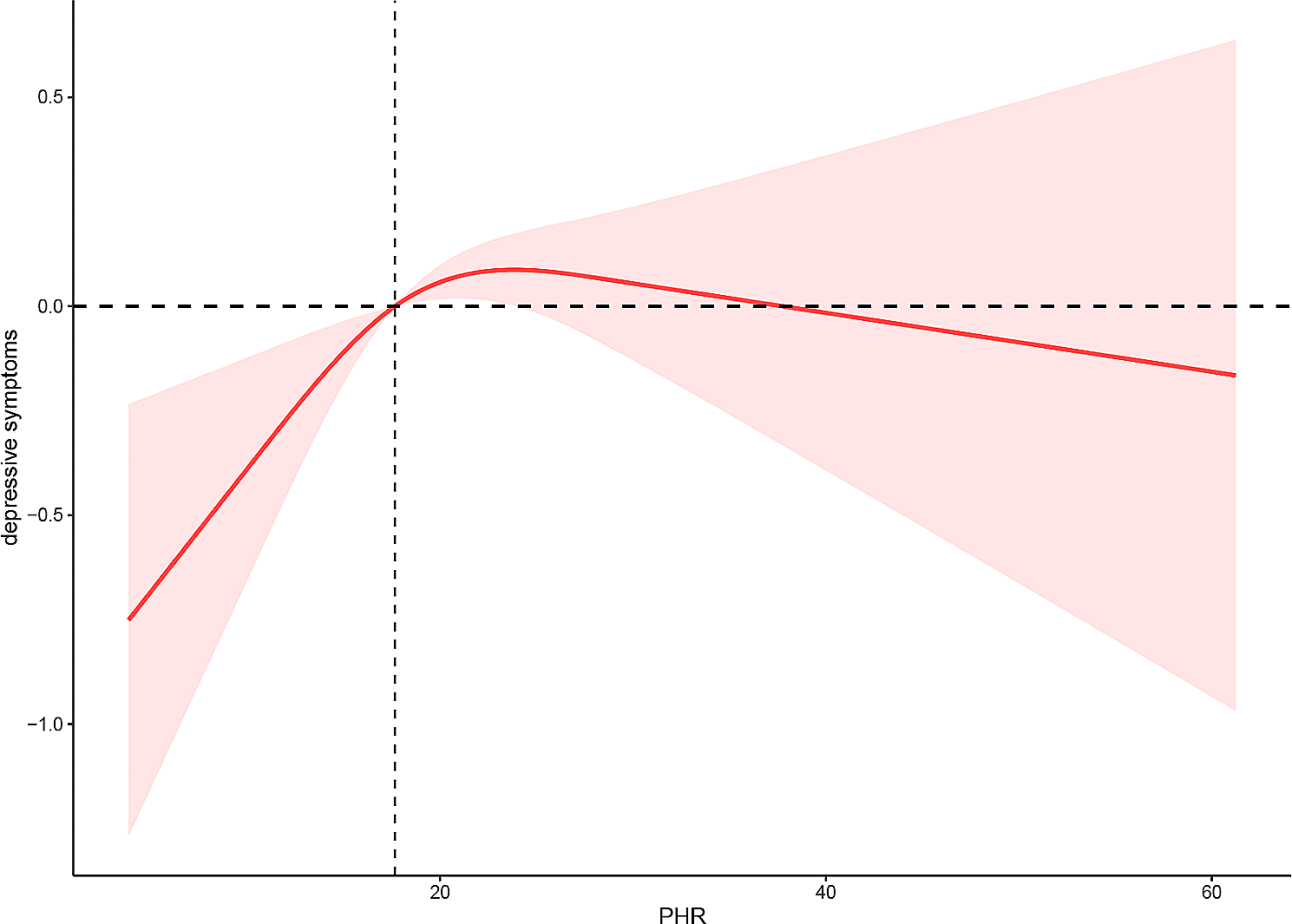
Restricted cubic spline analysis of the association between PHR values and depressive symptoms. Age, gender, race, education level, BMI (body mass index), marital status, family PIR (body mass index), smoking status, alcohol use, recreational activities, diet status, sleep disorder, DM (diabetes mellitus), hypertension, CKD (chronic kidney disease), ASCVD (arteriosclerotic cardiovascular disease), TC (fasting total cholesterol), LDL-C (low-density lipoprotein cholesterol) were all adjusted. OR, odds ratio; CI, confidence interval; Ref, reference

### Definition of PHR Index

Herein, PHR served as the exposure variable. Given that PHR values have considerable variability, PHR transformation was used instead. To calculate PHR index, we divided the platelet counts by HDL-C content, then divided the quotient by 10 [18].

### Assessment of depression symptoms

Depressive symptoms evaluation in NHANES utilized the 2007–2018 Patient Health Questionnaire-9 (PHQ-9). PHQ-9 (consisting of 9 items) was employed for depression screening in primary care settings, using a 4-point scale to quantify the responses (not at all = 0, several days = 1, over half of all days = 2, almost daily = 3). To compute the PHQ-9 score, all 9 items were added; elevated scores represented more severe depression symptoms; whereas, 0 and 27 were the minimum and maximum scores, respectively. Depression status was categorized into 4 groups: mild [5–9], moderate [10–14], moderate to severe [15–19], and severe [20–27]. A binary variable indicating no depression (PHQ-9 score < 10) or elevated depressive symptoms (PHQ-9 score ≥ 10) was created using a threshold score of 10; with 88% sensitivity and specificity [31–33]. The employed questionnaire can be accessed at https://wwwn.cdc.gov/Nchs/Nhanes/2013-2014/DPQ_H.htm.

### Biochemical parameters

Blood samples were collected for biochemical assessment at a mobile examination center (MEC). We analyzed platelet counts, HDL-C, low-density lipoprotein cholesterol (LDL-C), and fasting total cholesterol (TC) using standard protocols, as described previously [34, 35].

### Other covariates of interest

Herein, we evaluated a series of possible confounding factors highlighted by epidemiological research and associated with depressive symptoms. Among these factors were sociodemographic variables, namely, marital status, education status, family poverty-to-income ratio (PIR), race, gender and age; as well as physical variables, such as, body mass index (BMI); and lifestyle variables, including alcohol intake, smoking habit, recreational activities and diet status. Additionally, comorbid conditions namely diabetes mellitus (DM), hypertension, chronic kidney disease (CKD), arteriosclerotic cardiovascular disease (ASCVD) and sleep disorder was also investigated.

Attained through self-reported questionnaires, the sociodemographic information underwent intricate categorization. Age delineations included < 45, 45–64, or ≥ 65 years, while race/ethnicity options encompassed non-Hispanic black, non-Hispanic white, Mexican-American, or other, accommodating individuals with multiracial backgrounds. Marital status featured never married, married, or living separately, with divorced, widowed, or separately living individuals grouped under the category of those residing in individual households. Educational status was stratified into below high school (HS), HS graduate, or beyond HS, providing a comprehensive framework for sociodemographic characterization. WHO guidelines were used that BMI ≥ 25 kg/m^2 were designated as overweight, and this included obese individuals. The family PIR, when less than 1.00, indicates that the household income is below the poverty threshold, while a PIR exceeding 3.00 signifies that the income is more than three times the poverty threshold.

The assessment of lifestyle factors was undertaken through the administration of self-reported questionnaires as data collection’s an integral component. Alcohol intake was categorized as follows: lifetime abstainers, consumed < 12 drinks in lifetime; former drinkers, consumed ≥ 12 drinks in lifetime, but no intake in the last year before study; current light drinkers, consumed < 3 drinks each week; and current heavy drinkers, consumed > 3 drinks each week. Based on the NCHS and CDC, patient smoking habit was grouped as follows: never consumed or consumed < 100 cigarettes in lifetime; consumed ≥ 100 cigarettes but quit before interview; current consumers. Vigorous recreational activities were stratified as “yes” or “no” [36]. According to the United States Department of Agriculture (USDA) stratification guidelines, we adjusted the Healthy Eating Index (HEI) total score thresholds as 60, 70, and 80, respectively [37], with > 80 representing “good diet” and **≤** 80 representing “needs improvement”.

Among the analyzed comorbidities were hypertension, DM, CKD, ASCVD and sleep disorder. Hypertension inclusion criteria were hypertension diagnosis, anti-hypertensive drug usage, or systolic blood pressure (BP) ≥ 140 mmHg and/or diastolic BP ≥ 90 mmHg. Type 2 DM utilized the following criteria: a diabetes diagnosis, oral glucose tolerance ≥ 11.1 mmol/L, random glucose content ≥ 11.1 mmol/L, fasting glucose content ≥ 7.0 mmol/L, Hemoglobin A1c (HbA1c) ≥ 6.5%, or antidiabetic drug usage. The Chronic Kidney Disease Epidemiology Collaboration (CKD-EPI) formula was employed to assess the estimated glomerular filtration rate (eGFR) for CKD diagnosis, with CKD indicating eGFR < 60 ml/min/1.73 m^2, or a urine albumin-to-creatinine ratio (UACR) > 30 mg/g [38]. ASCVD and sleep disorder were assessed via self-reported data, with a ‘Yes’ or ‘No’ response.

### Statistical analysis

All data analyses employed R 4.2. Following NHANES guidelines, sampling weights were employed to diminish the decisive oversampling of specific demographics. All examinations were weighted for sample size and considered the intricate classified, multistep, cluster sampling approach employed in NHANES [39]. It was attempted to express continuous data in form of mean ± standard error (SE), incorporating adjustments for survey weights, while expression of categorical data was undertaken in form of count and percentage, similarly considering survey weight adjustments. Moreover, herein, we converted the PHR variable from continuous to a categorical scale. Thereafter, several models were developed to assess the individual impacts of PHR and depression symptoms on the outcome of interest. PHR was analyzed as continuous and as categorical variables based on quartiles. The evaluation of inter-group differences, predicated on both PHR quartiles and depressive symptoms, entailed the utilization of the weighted Chi-square test particularly for categorical data and the weighted Student’s t-test distinctly for continuous data.

We also utilized weighted uni- and multivariate logistic regression analyses to examine PHR and depressive symptoms relationship across different models. Crude Model was without flexible adjustments. Model 1 underwent adjustments for patient age, gender and race. Model 2 underwent adjustments for patient age, gender, race, BMI, marital status, family PIR, education status, smoking habit, alcohol intake, recreational activities, diet status, hypertension, CKD, ASCVD, DM, sleep disorder, LDL-C and TC. The *P*-value for the trend was established using a logistic regression model. Interaction assessments examined association heterogeneity among distinct subgroups. A restricted cubic spline (RCS) model containing three knots explored possible linear and non-linear associations. Knot number 3 selection was completed by minimizing the Akaike information criterion (AIC) statistic. Nonlinear data was assessed with a two-wise linear regression model (segmented regression model), fitting individual intervals while quantifying cut-off influences. The log-likelihood ratio test assessed linear or non-linear associations. Significance cut-off was at *p*-value < 0.05.

### Logistic regression analyses

Using multivariate analysis (Table 2), we exhibited a strong association between exposure and outcome variables, which remained post adjustment of confounding factors (*P-*value < 0.05). When treated as continuous data, we only identified a significantly direct association between PHR and the likelihood of elevated depressive symptoms in crude model (odds ratio [OR] = 1.02, 95% confidence interval [CI]: 1.01–1.03, *P* < 0.0001) and model 1 (OR = 1.02, 95% CI: 1.02– 1.03, *P* < 0.0001). However, no marked relationship was seen between the PHR quartiles and elevated depressive symptoms in the fully adjusted Model 2. As categorical data (divided into quartiles), the largest PHR quartile was directly and significant linked to elevated depressive symptoms relative to the minimum quartile across all models. Especially in fully adjusted model 2, we observed that those with the fourth quartile experienced a drastically raised depressive symptoms by 53% (OR = 1.53, 95% CI: 1.00–2.33, *P* = 0.05) relative to those with the smallest PHR quartile. However, the *P*-value for the trend across quartiles was 0.06 in fully adjusted model 2. Additionly, the results of our univariate analysis was shown in Supplementary Table 2

**Table 2:** Relationship between PHR and depressive symptoms, showed by weighted multivariate logistic regression

	Crude model		Model 1		Model 2	
**Depressive symptoms**	OR (95% CI)	*P*-value	OR (95% CI)	*P*-value	OR (95% CI)	*P*-value
**PHR continuous**	1.02(1.01,1.03)	< 0.0001	1.02(1.02,1.03)	< 0.0001	1.01(1.00,1.03)	0.13
**PHR categories**						
Q1	ref		ref		ref	
Q2	1.31(1.10,1.57)	0.003	1.38(1.15,1.65)	< 0.001	1.65(1.06,2.55)	0.03
Q3	1.49(1.27,1.76)	< 0.0001	1.60(1.35,1.89)	< 0.0001	1.66(1.11,2.48)	0.02
Q4	1.71(1.45,2.01)	< 0.0001	1.83(1.54,2.17)	< 0.0001	1.53(1.00,2.33)	0.05
*P* for trend		< 0.0001		< 0.0001		0.06

Crudel model: PHR (platelet/high-density lipoprotein cholesterol ratio). Model 1: PHR, gender, age, race. Model 2: PHR, age, gender, race, BMI (body mass index), marital status, education level, family PIR (poverty income ratio), smoking status, DM (diabetes mellitus), alcohol consumption, recreational activities, diet status, sleep disorder, hypertension, ASCVD (arteriosclerotic cardiovascular disease), CKD (chronic kidney disease), TC (fasting total cholesterol), LDL-C (low-density lipoprotein cholesterol). OR; odds ratio, CI; confidence interval, Ref; reference

After full adjustments, we observed an inverted U-shaped relation between PHR and depressive symptoms (Fig. 2). Accordingly, we carried out a two-piece-wise regression model assessment and discovered that this model performed superior to the nonlinear model in explaining the crucial link between PHR and depressive symptoms (log-likelihood ratio (LLR) = 0.06, Table 3). The critical PHR value was 23.76, with PHR range between 0–23.76, PHR increased by 1 unit, the depressive symptoms raised by 0.06 (OR = 1.06, 95% CI: 1.02–1.10, *P* = 0.01). However, when the range of PHR was ≥ 23.76, the link between PHR and depressive symptoms was insignificant (OR = 1.00, 95% CI: 0.96–1.03, *P* = 0.85)

**Table 3:** Results of binary logistic regression and piecewise linear regression model

Outcome: elevated depressive symptoms	Adjusted OR (95% CI)	*P*–value
Fitting by binary logistic regression model	1.01(1.00, 1.03)	0.13
Fitting by the two–piecewise linear model		
Inflection point	23.76	
PHR < 23.76	1.06(1.02, 1.10)	0.01
PHR ≥ 23.76	1.00(0.96, 1.03)	0.85
Log–likelihood ratio	0.06	

All models were adjusted for age, gender, race, education level, BMI (body mass index), marital status, family PIR (body mass index), smoking status, alcohol use, recreational activities, diet status, sleep disorder, DM (diabetes mellitus), hypertension, CKD (chronic kidney disease), ASCVD (arteriosclerotic cardiovascular disease), TC (fasting total cholesterol), LDL-C (low-density lipoprotein cholesterol). OR, odds ratio; CI, confidence interval

### Stratified assessment

Through stratified analysis, each sample in the study was categorized and analyzed independently to ascertain the influence of confounding factors and specific population. As depicted in Table 4, the data suggest that apart from Mexican American, individuals who are never married, those with a history of current or former smoking, and those with a history of former alcohol use, as well as individuals who engage in vigorous recreational activities, have a good diet, or suffer from sleep disorders (*P* > 0.05), the majority of demographic groups could be a significantly relevant population that is particularly vulnerable to the impact of PHR, which might lead to an increase in depressive symptoms (*P* < 0.05)

Furthermore, we assessed associations with several patient characteristics—age, gender, race, BMI, marital status, family PIR, education level, smoking status, alcohol intake, recreational activities, diet status, hypertension, CKD, ASCVD, DM and sleep disorders—determine the possibility of strong dependence of the effect modifier on this association. In this report, the association reached significance only amongst gender- and alcohol consumption-stratified population (*P* for interaction < 0.05), indicating that gender and alcohol use may modify the relationship between PHR and depression symptoms

**Table 4:** Subgroup analyses of the relationship between PHR values and the risk of depressive symptoms

Characteristics	Q1	Q2	Q3	Q4	*P* for trend	*P* for interaction
Age (years)						0.907
<45	ref	1.210(0.928,1.577)	1.403(1.043,1.889)	1.611(1.228,2.115)	< 0.001	
45–64	ref	1.258(0.926,1.709)	1.402(1.054,1.866)	1.632(1.230,2.166)	< 0.001	
≥65	ref	1.607(1.131,2.283)	1.759(1.257,2.460)	1.764(1.176,2.646)	0.001	
Sex						0.002
Male	ref	0.963(0.703,1.320)	1.027(0.762,1.385)	1.289(1.003,1.657)	0.038	
Female	ref	1.655(1.325,2.068)	2.067(1.686,2.534)	2.269(1.846,2.788)	< 0.0001	
Race						0.124
Mexican American	ref	1.495(1.075,2.078)	1.359(0.956,1.932)	1.303(0.908,1.868)	0.476	
Non-Hispanic Black	ref	1.323(1.019,1.718)	1.350(1.043,1.748)	1.523(1.146,2.025)	0.003	
Non-Hispanic White	ref	1.404(1.100,1.791)	1.548(1.208,1.983)	1.806(1.448,2.252)	< 0.0001	
Other race	ref	0.854(0.561,1.299)	1.409(0.944,2.104)	1.645(1.105,2.449)	0.002	
Education level						0.104
Less than high school	ref	1.897(1.278,2.816)	1.912(1.220,2.999)	2.054(1.378,3.060)	0.003	
High school	ref	1.050(0.825,1.338)	1.076(0.838,1.382)	1.391(1.076,1.799)	0.012	
More than high school	ref	1.415(1.051,1.904)	1.692(1.307,2.191)	1.696(1.347,2.135)	< 0.0001	
Marital status						0.19
Married	ref	1.396(1.104,1.766)	1.592(1.204,2.106)	2.059(1.560,2.717)	< 0.0001	
Live separated	ref	1.297(0.999,1.685)	1.444(1.117,1.867)	1.702(1.294,2.238)	< 0.001	
Never married	ref	1.239(0.847,1.813)	1.484(1.077,2.044)	1.247(0.899,1.731)	0.11	
Family PIR						0.517
< 1	ref	1.243(0.922,1.676)	1.234(0.923,1.650)	1.488(1.157,1.915)	0.003	
1–3	ref	1.071(0.777,1.475)	1.232(0.972,1.561)	1.353(1.044,1.753)	0.008	
> 3	ref	1.646(1.156,2.344)	1.670(1.083,2.575)	1.643(1.076,2.508)	0.025	
BMI (kg/m^2)						0.661
<25	ref	1.232(0.923,1.643)	1.415(1.054,1.899)	1.911(1.394,2.620)	< 0.0001	
≥25	ref	1.335(1.062,1.677)	1.475(1.203,1.807)	1.640(1.350,1.993)	< 0.0001	
Smoking status						0.091
Never	ref	1.510(1.161,1.964)	1.806(1.413,2.307)	1.882(1.428,2.481)	< 0.0001	
Former	ref	1.071(0.740,1.550)	1.312(0.970,1.774)	1.248(0.885,1.758)	0.077	
Now	ref	1.182(0.946,1.477)	1.064(0.817,1.384)	1.311(1.028,1.672)	0.082	
Alcohol usage						0.029
Never	ref	1.548(1.002,2.392)	2.412(1.588,3.662)	2.175(1.394,3.393)	< 0.0001	
Former	ref	1.006(0.711,1.423)	1.247(0.905,1.718)	1.159(0.845,1.589)	0.209	
Moderate	ref	1.739(1.286,2.352)	2.055(1.432,2.947)	2.338(1.687,3.241)	< 0.0001	
Heavy	ref	1.063(0.771,1.465)	1.115(0.813,1.528)	1.419(1.070,1.884)	0.024	
Vigorous recreational activities						0.786
No	ref	1.311(1.099,1.565)	1.467(1.239,1.737)	1.617(1.361,1.923)	< 0.0001	
Yes	ref	1.164(0.782,1.732)	1.187(0.758,1.858)	1.343(0.932,1.935)	0.156	
Diet status						0.618
Needs improvement	ref	1.301(1.079,1.570)	1.471(1.239,1.747)	1.703(1.437,2.018)	< 0.0001	
Good diet	ref	1.092(0.330,3.615)	1.886(0.669,5.318)	0.716(0.114,4.495)	0.636	
DM						0.776
No	ref	1.266(1.037,1.547)	1.473(1.218,1.781)	1.612(1.340,1.939)	< 0.0001	
Yes	ref	1.451(0.987,2.133)	1.414(1.003,1.994)	1.719(1.250,2.364)	0.005	
Hypertension						0.534
No	ref	1.234(0.979,1.556)	1.533(1.212,1.939)	1.605(1.267,2.035)	< 0.0001	
Yes	ref	1.434(1.116,1.842)	1.441(1.139,1.824)	1.766(1.394,2.238)	< 0.0001	
CKD						0.835
No	ref	1.326(1.081,1.625)	1.518(1.245,1.851)	1.703(1.409,2.059)	< 0.0001	
Yes	ref	1.383(1.012,1.891)	1.405(1.005,1.964)	1.846(1.405,2.426)	< 0.0001	
ASCVD						0.053
No	ref	1.322(1.094,1.597)	1.547(1.290,1.855)	1.639(1.382,1.943)	< 0.0001	
Yes	ref	1.333(0.915,1.943)	1.251(0.878,1.781)	2.208(1.451,3.360)	< 0.001	
Sleep disorder						0.33
No	ref	1.337(1.086,1.647)	1.533(1.211,1.941)	1.676(1.356,2.072)	< 0.0001	
Yes	ref	0.943(0.564,1.576)	1.003(0.630,1.597)	1.277(0.861,1.894)	0.128	

Abbreviations: PHR, platelet/high-density lipoprotein cholesterol ratio; BMI, body mass index; PIR, poverty income ratio; DM, diabetes mellitus; CKD, chronic kidney disease; ASCVD, arteriosclerotic cardiovascular disease; OR, odds ratio; CI confidence interval; Ref, reference

## Discussion

We examined the association between PHR and depressive symptoms in this population-based cross-sectional study. Higher PHR index was associated with a higher prevalence of depressive symptoms in the included US adult population, even after adjusting for potential confounders. Additionally, this association remained significant in subgroup analyses. In the RCS analysis, PHR demonstrated a pronounced non-linear association with the risk of elevated depressive symptoms, providing substantial evidence for further clinical and basic research

PHR is a novel combination indicator that evaluates systematic inflammatory of the human body. Despite no prior reports of a link between PHR and depressive symptoms, the relation between HDL-C and depression is widely studied. Based on a cross-sectional investigation involving 64 subjects, circulating HDL-C content is markedly diminished among major depression sufferers [26]. Similar to our conclusions, one prospective investigation revealed that people suffering from anxiety or depression exhibit reduced HDL-C contents, relative to normal controls [40]. According to a Mendelian randomization investigation, diminished HDL-C levels were intricately linked to enhanced depressive symptom risks (OR 2.17, 95% CI 1.40–3.39) [41]. Although, as already mentioned, some earlier studies corroborated with our results, other investigations produced alternate results. For instance, a cross-sectional investigation involving 870 Chinese elderly individuals demonstrated no marked association between HDL-C levels and depression risk [42]. This corroborated another study involving elderly population that also demonstrated no link between the two aforementioned variables [43]. The KNHANES investigation, involving Korean middle-aged adults, reported augmented HDL-C levels among individuals with enhanced depressive symptom risks [44]. Discrepancy in the aforementioned investigations may be due to inherent alterations in subject ethnicity and depression evaluation. Hence, it is crucial to employ reliable inflammatory parameters to enhance elucidation of a potential HDL-C and depression link. In our analyses, the results from the continuous model of PHR and the categorical model were inconsistent. Although the direction for correlation is positive, the results should be interpreted with caution. Based on subcategorical evaluation, subgroup classification by gender and alcohol intake have a strong association with PHR. A recent cross-sectional study in Germany revealed that augmented depressive symptom severity was obvious among medical care patients who consume large quantities of alcohol. Moreover, using interaction analyses, it was revealed that depression risk was steeper among women and younger individuals who consume excess alcohol [45]. Therefore, the mediation effect of gender and alcohol intake in relation PHR and depressive symptoms risk warrants additional exploration

There are limited reports on the associated signaling networks connecting lipid profiles to depression. Cholesterol is a critical modulator of neurological development, and it strictly controls membrane-integrated proteins, ion channels, and synapse formation. One early investigation demonstrated a strong inverse relation between cholesterol and suicidality via the serotonergic (5-HT) system. Circulating cholesterol is also correlated with a transformed inflammatory distribution [46]. Lipid profile dysregulation is common in metabolic syndrome. Prior investigation revealed that inflammatory activation and augmented oxidative stress are common pathways that regulate both metabolic syndrome and depression [3]. GWAS summary data assessment further revealed that cardiometabolic features, such as, HDL-C, LDL-C, TC, and TG exhibit over-lapping genomic loci with depression, which also support the presence of comparable signaling pathways [41]. One review concluded the existence of bi-directional control between HDL-C content and depression risk. Emerging evidences suggest that reduced HDL-C is common among depression sufferers. Alternately, diminished HDL-C content activates inflammatory and oxidative processes to accelerate depression development. In a physiological state, HDL-C removes circulating LPS to suppress macrophage and lymphocyte maturation and activation to reduce inflammation. HDL-C also serves as an antioxidant by downregulating lipid peroxidation while maintaining mitochondrial energy synthesis [21]. Nonetheless, reduced HDL-C content among depression patients dampens these protective influences. The signaling responsible for the initial decline of HDL-C among depression patients warrants further analyses. The NHANES study reported large quantities of proinflammatory cytokines, namely, IL-6, IL-1, and TNF- α, within depressed individuals, which corroborates with a previously proposed signaling network [47]. Herein, we revealed a strong relation between PHR and enhanced depression risk, which is also indicative of a negative reduced HDL-C effect. However, the associated signaling network remains unelucidated and warrants additional exploration

Platelets are non-specific inflammatory indicators that physically associate with leukocytes and the endothelium to modulate inflammatory activity, such as that of cytokines, epinephrine, serotonin, glutamate, dopamine, and P-selectin, within these cells [48]. Serotonin, glutamate, and other proinflammatory molecules, namely, IL-1, CD40L, and P-selectin are secreted from activated platelets, and these, in turn, refulate platelet activity in depressed individuals [49, 50]. Platelets possess glutamate-harboring dense granules [48, 51], and depression is reported to activate platelets. Herein, we revealed that augmented platelet contents are a stand-alone risk factor for depression status

Depression-related risk factors reported previously include obesity, smoking, sleep disorder, DM, hypertension, hyperlipidemia, CKD and ASCVD [52–55]. A recent systematic review and meta-analysis reported that physically active adults experience reduced depression risk, relative to physically inactive adults [56]. Here, individuals exhibiting symptoms consistent with mild-to-severe depression tended to exhibit a lower education level and household income as compared to subjects without depression. This may indicate that reduced education and household income status are intricately linked to a less healthy lifestyle that includes higher rates of obesity, poor diet, inactivity, smoking, and a failure to comply with medication [57, 58]. Lower levels of education and the consequences thereof may thus partially account for the detected link between inflammation and depression

This investigation has multiple notable strengths. Firstly, we analyzed NHANES information, and suitable NHANES sample weights were fully counted for. Secondly, confounding factors were thoroughly adjusted to increase conclusion reliability and to enable generalizability to a larger population. Thirdly, routine blood and blood biochemistry evaluation provided a cost-effective, simplistic, and extensive information for the diagnosis and management of individuals with elevated depressive symptoms. Therefore, this approach required additional investigation and analyses

Nonetheless, we must discuss the limitations of this investigation. Firstly, due to the design of a cross-sectional study, we were unable to evaluate the causality between PHR and depressive symptoms. Thus, reverse causality may also occur because of the bi-directional relationship between PHR and depressive symptoms. Secondly, while PHQ-9 is a validated screening tool for assessing the frequency of depressive symptoms, it is not suitable for diagnosing clinical depression. Notably, the NHANES database did not include detailed clinical variables such as personal medication histories (both psychotropic and somatic) or comorbid psychiatric disorders (i.e., anxiety and substance use), both of which warrant further investigation. Several investigations, however, have demonstrated strong specificity and sensitivity in PHQ-9-based major depression diagnosis, which may reduce potential assessment errors in our investigation [32, 33]. Additionally, we used NHANES blood samples data from a single blood extraction. Sequential testing could potentially be more indicative of true physiological states, given the lifespan of blood cells. Thirdly, the database has its own internal limitations, such as using self-reported questionnaire to assess other covariates rather than diagnostic interviews for objective measures, which may have introduced confounding factors that influenced the results. Fourthly, given the randomly missing data among the covariables and the large sample size, the study refrained from employing multiple imputation methods to handle the missing data, which may affect the precision of the findings. However, this approach may potentially impact the accuracy of the findings. Lastly, although we detected a statistically significant association between depressive symptoms and PHR, the effect magnitude was very small and therefore may not reflect a clinically relevant relationship

## Conclusion

In summary, herein, we revealed a positive link between raised PHR and elevated depressive symptoms risk among US adults when PHR < 23.76. Additional prospective investigations and randomized controlled trials are warranted to validate our findings. The underlying mechanism and potential therapeutic effect still require more investigation

### Electronic supplementary material

Below is the link to the electronic supplementary material.

Supplementary Material 1

Supplementary Material 2

## Data Availability

The datasets generated and analyzed during the current study are available in the NHANES repository, https://www.cdc.gov/nchs/nhanes/.
