# Supplementary material for: Examining the cross-sectional relationship of platelet/high-density lipoprotein cholesterol ratio with depressive symptoms in adults in the United States
Source: BMC Psychiatry. 2024 Jun 7;24:427. doi: 10.1186/s12888-024-05878-x (PMC11157938; doi:10.1186/s12888-024-05878-x)
Supplement: Supplementary file 1 — Supplementary Material 1 [file 12888_2024_5878_MOESM1_ESM.doc]

| **Supplementary Table 1 Baseline characteristics of the study population** | | | | | | |
| --- | --- | --- | --- | --- | --- | --- |
| **Characteristics (weighted)** |  | **PHR categories** | | | |  |
|  | **Total (N = 28098)** | **Q1(≤13.95)** | **Q2 >13.95, ≤18.15** | **Q3 > 18.15, ≤23.47** | **Q4 >23.47** | ***P*-value** |
| Platelet (1000cells/uL) | 243.43±0.85 | 197.25±0.87 | 228.81±0.72 | 253.02±0.94 | 297.56±1.32 | < 0.0001 |
| HDL-C (mmol/L) | 1.38±0.01 | 1.82±0.01 | 1.43±0.00 | 1.23±0.00 | 1.01±0.00 | < 0.0001 |
| LDL-C (mmol/L) | 2.94±0.01 | 2.80±0.02 | 2.96±0.02 | 3.06±0.02 | 2.98±0.02 | < 0.0001 |
| Fasting TC (mmol/L) | 5.00±0.01 | 5.07±0.02 | 4.96±0.02 | 4.99±0.02 | 4.97±0.02 | < 0.001 |
| Elevated depressive symptoms, n (%) |  |  |  |  |  | < 0.0001 |
| No | 25508(91.97) | 6534(94.01) | 6406(92.28) | 6347(91.31) | 6221(90.19) |  |
| Yes | 2590(8.03) | 493(5.99) | 612(7.72) | 683(8.69) | 802(9.81) |  |
| Depression severity, n (%) |  |  |  |  |  | < 0.0001 |
| None | 21020(76.56) | 5516(79.71) | 5364(78.59) | 5119(74.16) | 5021(73.58) |  |
| Mild | 4488(15.41) | 1018(14.29) | 1042(13.69) | 1228(17.15) | 1200(16.61) |  |
| Moderate | 1603(5.06) | 324(4.08) | 390(5.04) | 412(5.30) | 477(5.88) |  |
| Moderately-Severe | 699(2.12) | 120(1.35) | 169(2.00) | 185(2.31) | 225(2.85) |  |
| Severe | 288(0.85) | 49(0.56) | 53(0.68) | 86(1.08) | 100(1.08) |  |
| Age (years, n (%)) |  |  |  |  |  | < 0.0001 |
| <45 | 11370(44.09) | 2206(34.47) | 2743(43.41) | 2986(47.16) | 3435(51.74) |  |
| 45-64 | 9906(36.95) | 2442(38.12) | 2435(36.34) | 2556(37.08) | 2473(36.22) |  |
| ≥65 | 6822(18.96) | 2379(27.41) | 1840(20.24) | 1488(15.76) | 1115(12.04) |  |
| Sex, n (%) |  |  |  |  |  | < 0.0001 |
| Male | 13977(49.37) | 3075(39.86) | 3443(49.08) | 3697(53.81) | 3762(55.07) |  |
| Female | 14121(50.63) | 3952(60.14) | 3575(50.92) | 3333(46.19) | 3261(44.93) |  |
| Race, n (%) |  |  |  |  |  | < 0.0001 |
| Mexican American | 4287(8.53) | 743(5.43) | 1009(7.85) | 1212(9.69) | 1323(11.32) |  |
| Non-Hispanic Black | 5754(10.44) | 1773(11.92) | 1437(10.24) | 1327(9.84) | 1217(9.73) |  |
| Non-Hispanic White | 11898(67.73) | 3116(71.62) | 3005(69.10) | 2901(66.26) | 2876(63.72) |  |
| Other race | 6159(13.30) | 1395(11.02) | 1567(12.82) | 1590(14.22) | 1607(15.24) |  |
| Marital status, n (%) |  |  |  |  |  | 0.001 |
| Married | 16689(63.41) | 3963(63.17) | 4218(63.33) | 4246(63.32) | 4262(63.86) |  |
| Live separated | 6303(18.49) | 1831(20.48) | 1556(18.59) | 1474(17.56) | 1442(17.24) |  |
| Never married | 5093(18.07) | 1231(16.34) | 1240(18.02) | 1306(19.08) | 1316(18.89) |  |
| Missing | 13(0.03) | 2(0.01) | 4(0.07) | 4(0.03) | 3(0.01) |  |
| Education level, n (%) |  |  |  |  |  | < 0.0001 |
| Less than high school | 2787(4.94) | 592(3.99) | 654(4.51) | 752(5.29) | 789(6.04) |  |
| High school | 10332(33.37) | 2317(28.27) | 2518(31.83) | 2661(35.66) | 2836(38.01) |  |
| More than high school | 14958(61.64) | 4113(67.68) | 3844(63.65) | 3610(58.99) | 3391(55.89) |  |
| Missing | 21(0.04) | 5(0.05) | 2(0.01) | 7(0.06) | 7(0.06) |  |
| Family PIR, n (%) |  |  |  |  |  | < 0.0001 |
| < 1 | 5358(13.01) | 1137(10.16) | 1246(12.42) | 1403(13.50) | 1572(16.14) |  |
| 1-3 | 10931(33.50) | 2619(30.42) | 2625(31.87) | 2798(35.40) | 2889(36.51) |  |
| > 3 | 9328(46.42) | 2639(52.22) | 2509(48.82) | 2228(43.67) | 1952(40.63) |  |
| Missing | 2481(7.06) | 632(7.20) | 638(6.89) | 601(7.43) | 610(6.72) |  |
| BMI (kg/m^2, n (%)) |  |  |  |  |  | < 0.0001 |
| <25 | 7778(28.77) | 3057(45.86) | 2207(32.15) | 1522(22.28) | 992(13.94) |  |
| ≥25 | 20061(70.55) | 3907(53.54) | 4750(67.12) | 5458(77.21) | 5946(85.20) |  |
| Missing | 259(0.67) | 63(0.60) | 61(0.73) | 50(0.51) | 85(0.87) |  |
| Smoking status, n (%) |  |  |  |  |  | < 0.0001 |
| Never | 15479(55.22) | 4015(57.54) | 3994(57.03) | 3859(54.31) | 3611(51.82) |  |
| Former | 6877(25.17) | 1843(27.53) | 1746(25.54) | 1707(24.81) | 1581(22.66) |  |
| Now | 5725(19.57) | 1162(14.85) | 1276(17.42) | 1462(20.87) | 1825(25.45) |  |
| Missing | 17(0.04) | 7(0.08) | 2(0.01) | 2(0.01) | 6(0.07) |  |
| Alcohol usage, n (%) |  |  |  |  |  | < 0.0001 |
| Never | 3869(10.47) | 942(9.99) | 960(10.24) | 967(10.45) | 1000(11.26) |  |
| Former | 4240(12.31) | 936(9.55) | 942(11.04) | 1117(13.58) | 1245(15.26) |  |
| Moderate | 6722(27.52) | 2064(35.99) | 1778(28.98) | 1530(23.99) | 1350(20.73) |  |
| Heavy | 5546(20.97) | 1245(19.58) | 1361(20.09) | 1406(21.46) | 1534(22.86) |  |
| Missing | 7721(28.72) | 1840(24.89) | 1977(29.66) | 2010(30.52) | 1894(29.89) |  |
| Vigorous recreational activities, n (%) |  |  |  |  |  | < 0.0001 |
| No | 21913(73.73) | 5245(68.94) | 5366(71.69) | 5545(75.23) | 5757(79.40) |  |
| Yes | 6185(26.27) | 1782(31.06) | 1652(28.31) | 1485(24.77) | 1266(20.60) |  |
| Diet status, n (%) |  |  |  |  |  | < 0.0001 |
| Needs improvement | 26476(95.13) | 6539(93.86) | 6572(94.53) | 6642(95.85) | 6723(96.37) |  |
| Good diet | 624(2.16) | 220(3.40) | 181(2.56) | 133(1.69) | 90(0.92) |  |
| Missing | 998(2.71) | 268(2.74) | 265(2.91) | 255(2.47) | 210(2.71) |  |
| Sleep disorder, n (%) |  |  |  |  |  | < 0.0001 |
| No | 17292(58.88) | 4206(56.98) | 4338(59.22) | 4331(59.22) | 4417(60.17) |  |
| Yes | 1669(5.82) | 324(4.49) | 393(5.20) | 450(6.30) | 502(7.40) |  |
| Missing | 9137(35.29) | 2497(38.53) | 2287(35.58) | 2249(34.48) | 2104(32.42) |  |
| DM, n (%) |  |  |  |  |  | < 0.0001 |
| No | 22539(85.16) | 5919(89.39) | 5723(86.79) | 5575(84.31) | 5322(79.86) |  |
| Yes | 5559(14.84) | 1108(10.61) | 1295(13.21) | 1455(15.69) | 1701(20.14) |  |
| Hypertension, n (%) |  |  |  |  |  | 0.02 |
| No | 16240(62.87) | 3965(63.57) | 4171(64.75) | 4138(63.28) | 3966(59.74) |  |
| Yes | 11857(37.13) | 3062(36.43) | 2846(35.25) | 2892(36.72) | 3057(40.26) |  |
| Missing | 1(0.00) | 0(0.00) | 1(0.01) | 0(0.00) | 0(0.00) |  |
| CKD, n (%) |  |  |  |  |  | < 0.0001 |
| No | 22730(84.71) | 5540(82.93) | 5763(85.53) | 5815(86.59) | 5612(83.77) |  |
| Yes | 5105(14.49) | 1428(16.43) | 1187(13.67) | 1154(12.58) | 1336(15.28) |  |
| Missing | 263(0.80) | 59(0.64) | 68(0.80) | 61(0.83) | 75(0.95) |  |
| ASCVD, n (%) |  |  |  |  |  | 0.19 |
| No | 25244(91.86) | 6242(91.36) | 6316(92.12) | 6367(92.36) | 6319(91.59) |  |
| Yes | 2849(8.13) | 784(8.64) | 701(7.87) | 662(7.64) | 702(8.39) |  |
| Missing | 5(0.01) | 1(0.00) | 1(0.01) | 1(0.00) | 2(0.02) |  |
| Continuous data are shown as as means and standard error (SE), while categorical data are presented as percentages.  **Abbreviations:** HDL-C, high-density lipoprotein cholesterol; LDL-C, low-density lipoprotein cholesterol; TC, fasting total cholesterol; PIR, poverty income ratio; BMI, body mass index; PHR, platelet/high-density lipoprotein cholesterol ratio; CKD, chronic kidney disease; DM, diabetes mellitus; ASCVD, arteriosclerotic cardiovascular disease. | | | | | | |
