# Supplementary material for: Examining the cross-sectional relationship of platelet/high-density lipoprotein cholesterol ratio with depressive symptoms in adults in the United States
Source: BMC Psychiatry. 2024 Jun 7;24:427. doi: 10.1186/s12888-024-05878-x (PMC11157938; doi:10.1186/s12888-024-05878-x)
Supplement: Supplementary file 2 — Supplementary Material 2 [file 12888_2024_5878_MOESM2_ESM.doc]

| **Supplementary Table 2 Univariate logistic regression analysis of various variables** | | |
| --- | --- | --- |
| **Variables** | **OR (95% CI)** | ***P*-value** |
| Age (versus <45, years) |  |  |
| 45-64 | 1.16(1.03,1.30) | 0.01 |
| ≥65 | 0.75(0.62,0.90) | 0.003 |
| Sex (versus Male) |  |  |
| Female | 1.80(1.61,2.03) | <0.0001 |
| Race (versus Mexican American) |  |  |
| Non-Hispanic Black | 1.28(1.07,1.53) | 0.01 |
| Non-Hispanic White | 0.96(0.80,1.17) | 0.69 |
| Other race | 1.23(1.01,1.51) | 0.04 |
| BMI (versus < 25, kg/m^2) |  |  |
| ≥25 | 1.19(1.04,1.35) | 0.01 |
| Missing | 1.64(1.07,2.51) | 0.02 |
| Marital status (versus Married) |  |  |
| Live separated | 2.45(2.18, 2.74) | <0.0001 |
| Never married | 1.65(1.43, 1.90) | <0.0001 |
| Missing | 5.45(1.12,26.51) | 0.04 |
| Education level (versus Less than high school) |  |  |
| High school | 0.72(0.63,0.84) | <0.0001 |
| More than high school | 0.42(0.35,0.50) | <0.0001 |
| Missing | 0.94(0.15,5.82) | 0.95 |
| Family PIR (versus < 1) |  |  |
| 1-3 | 0.56(0.49,0.63) | <0.0001 |
| > 3 | 0.22(0.19,0.26) | <0.0001 |
| Missing | 0.45(0.37,0.55) | <0.0001 |
| Smoking status (versus Never) |  |  |
| Former | 1.31(1.11,1.54) | 0.002 |
| Now | 3.20(2.81,3.65) | <0.0001 |
| Missing | 0.00(0.00,0.00) | <0.0001 |
| Alcohol usage (versus Never) |  |  |
| Former | 1.79(1.49,2.15) | <0.0001 |
| Moderate | 1.14(0.96,1.36) | 0.13 |
| Heavy | 1.42(1.21,1.67) | <0.0001 |
| Missing | 0.77(0.66,0.91) | 0.003 |
| Vigorous recreational activities (versus No) |  |  |
| Yes | 0.35(0.29,0.42) | <0.0001 |
| Diet status (versus Needs improvement) |  |  |
| Good diet | 0.56(0.36,0.88) | 0.01 |
| Missing | 1.40(1.01,1.92) | 0.04 |
| DM (versus No) |  |  |
| Yes | 1.60(1.40,1.83) | <0.0001 |
| Hypertension (versus No) |  |  |
| Yes | 1.56(1.40,1.75) | <0.0001 |
| Missing | 0.00(0.00,0.01) | <0.0001 |
| CKD (versus No) |  |  |
| Yes | 1.30(1.16,1.46) | <0.0001 |
| Missing | 1.28(0.71,2.31) | 0.40 |
| ASCVD (versus No) |  |  |
| Yes | 2.13(1.83, 2.48) | <0.0001 |
| Missing | 4.78(0.48,48.08) | 0.18 |
| Sleep disorder (versus No) |  |  |
| Yes | 3.98(3.37,4.69) | <0.0001 |
| Missing | 1.22(1.06,1.40) | 0.01 |
| PHR continuous | 1.02(1.01,1.03) | <0.0001 |
| Platelet (1000cells/uL) | 1.00(1.00,1.00) | <0.0001 |
| HDL-C (mmol/L) | 0.73(0.64,0.83) | <0.0001 |
| LDL-C (mmol/L) | 1.05(0.95,1.17) | 0.32 |
| Fasting TC (mmol/L) | 1.06(1.00,1.11) | 0.04 |
| **Abbreviations:** HDL-C, high-density lipoprotein cholesterol; LDL-C, low-density lipoprotein cholesterol; TC, fasting total cholesterol; PIR, poverty income ratio; BMI, body mass index; PHR, platelet/high-density lipoprotein cholesterol ratio; CKD, chronic kidney disease; DM, diabetes mellitus; ASCVD, arteriosclerotic cardiovascular disease; OR, odds ratio; CI, confidence interval. | | |
